# Satellite-based assessment of electricity restoration efforts in Puerto Rico after Hurricane Maria

**DOI:** 10.1371/journal.pone.0218883

**Published:** 2019-06-28

**Authors:** Miguel O. Román, Eleanor C. Stokes, Ranjay Shrestha, Zhuosen Wang, Lori Schultz, Edil A. Sepúlveda Carlo, Qingsong Sun, Jordan Bell, Andrew Molthan, Virginia Kalb, Chuanyi Ji, Karen C. Seto, Shanna N. McClain, Markus Enenkel

**Affiliations:** 1 Earth from Space Institute, Universities Space Research Association, Columbia, Maryland, United States of America; 2 NASA Goddard Space Flight Center, Greenbelt, Maryland, United States of America; 3 Earth System Science Interdisciplinary Center, University of Maryland, College Park, Maryland, United States of America; 4 Science Systems and Applications, Inc., Lanham, Maryland, United States of America; 5 Earth Science Branch, NASA Marshall Space Flight Center, Huntsville, Alabama, United States of America; 6 Earth System Science Center, University of Alabama in Huntsville, Huntsville, Alabama, United States of America; 7 Biospheric Sciences Laboratory, NASA Goddard Space Flight Center, Greenbelt, Maryland, United States of America; 8 School of Electrical and Computer Engineering, Georgia Institute of Technology, Atlanta, Georgia, United States of America; 9 School of Forestry and Environmental Studies, Yale University, New Haven, Connecticut, United States of America; 10 Disasters Program, NASA Headquarters, Washington, District of Columbia, United States of America; 11 Harvard Humanitarian Initiative, Cambridge, Massachusetts, United States of America; University of California Santa Barbara, UNITED STATES

## Abstract

A real-time understanding of the distribution and duration of power outages after a major disaster is a precursor to minimizing their harmful consequences. Here, we develop an approach for using daily satellite nighttime lights data to create spatially disaggregated power outage estimates, tracking electricity restoration efforts after disasters strike. In contrast to existing utility data, these estimates are independent, open, and publicly-available, consistently measured across regions that may be serviced by several different power companies, and inclusive of distributed power supply (off-grid systems). We apply the methodology in Puerto Rico following Hurricane Maria, which caused the longest blackout in US history. Within all of the island’s settlements, we track outages and recovery times, and link these measures to census-based demographic characteristics of residents. Our results show an 80% decrease in lights, in total, immediately after Hurricane Maria. During the recovery, a disproportionate share of long-duration power failures (> 120 days) occurred in rural municipalities (41% of rural municipalities vs. 29% of urban municipalities), and in the northern and eastern districts. Unexpectedly, we also identify large disparities in electricity recovery between neighborhoods within the same urban area, based primarily on the density of housing. For many urban areas, poor residents, the most vulnerable to increased mortality and morbidity risks from power losses, shouldered the longest outages because they lived in less dense, detached housing where electricity restoration lagged. The approach developed in this study demonstrates the potential of satellite-based estimates of power recovery to improve the real-time monitoring of disaster impacts, globally, at a spatial resolution that is actionable for the disaster response community.

## Introduction

Electricity is inextricably linked to the health, well-being, and productivity of a society [1]. Without electricity, many of the most basic needs of humans—temperature control, access to functioning health facilities, illumination, and communication—are left unmet [2]. Restoring access to electricity after a disaster quickly is therefore essential, both to minimizing economic losses, and to bolstering the capacity of residents to endure changing and challenging environments.

When power is not restored quickly, catastrophic consequences can ensue. For instance, Hurricane Maria, which hit Puerto Rico in September of 2017, had sustained winds of 155 mph that impacted electricity access for 1.5 million grid-connected customers across the island. However, it was not only the scale of the impact, but the duration of service interruption that led to the storm’s historic losses. While 64 people died from the storm directly (i.e., via structural collapse, flying debris, floods and drownings), an estimated 700 to 8400 excess deaths were associated with lengthy power outages and disruptions in other basic services in Maria’s aftermath [3, 4]. Power outages can interrupt dialysis, ventricular assistance, and respiratory machines, can make it more difficult to access health records and store medicines (e.g. insulin, vaccines, and others that require refrigeration), and can disrupt communication to and treatment in healthcare facilities [4, 5].

On top of the loss of life and increased risk of morbidity, power outages halt major industrial, trade, service, and agricultural activities, causing significant economic damages. Data from various studies estimate that outages from severe weather alone, cost the US $18–70 billion dollars annually, depending on the impacts included [6–8]. Impacts can range from lost output and wages due to business interruptions, spoiled food, and production delays [8]. These costs from power outages are estimated to increase to $1.5-$3.4 trillion ($2015) by the middle of the century [9] as climate change heightens the frequency and intensity of severe weather.

To respond to power outage events and to mitigate their cascading social and economic costs, the disaster response community needs data about who has lost access to electricity, and for how long. Power outage and recovery data can inform many aspects of disaster response: efforts by utility companies to restore the power grid, by emergency management professionals to coordinate life-sustaining disaster assistance, by other infrastructure sectors (communications, financial, health care) whose services are interdependent with the electrical system, by insurance companies to assess damages and address claims, and by researchers identifying the risk predictors of sustained power outages and mapping out vulnerabilities to the power grid to prevent future outages.

Outage data must have four characteristics to be actionable to these groups. First, disaster response is urgent by nature, requiring immediate, real-time access to information to estimate damages and help direct resources [10]. Second, recovery can be a long-term process, requiring continuous (and archived) data collection to capture the evolving response effort. Third, disasters often impact an entire region, especially in the case of large-scale hazards like hurricanes and storms, so data that is consistently collected and comparable across counties, states, and even countries are needed. Fourth, disaster-related disruptions do not impact all populations evenly, and restoration efforts necessarily need to prioritize some areas over others (e.g. hospitals, police and fire stations, communications facilities). Spatially-explicit monitoring, available at the street level, is essential for identifying where to send aid, and for understanding distributional impacts–i.e., “who” has been most affected and what is the capacity of the affected communities to cope.

Currently available power outage and recovery data, collected from utilities, does not satisfy these four characteristics. Utility data is limited, both in its coverage [11], its consistency across different service areas [12, 13], and its availability in real-time and as archived data [14]. An interdisciplinary study by MIT on the state of the US electric grid found that “data on outages are neither comprehensive nor consistent… Most outages occur within distribution systems, but only 35 U.S. States require utilities to report data on [distribution outages]… it is accordingly impossible to make comprehensive comparisons across space or over time.” [14]. In middle and low-income countries, outage reports can be even more limiting as monitoring efforts are diminished by budget constraints. One study found that, on average for 109 developing countries, utilities reported 15% of the power outages that customers reported [15]. As, the frequency and intensity of severe weather events are predicted to increase with climate change [16], likely leading to larger and longer power disruptions, approaches that fill these data gaps, and can be applied both in the US and globally, will be critical to inform and implement effective recovery efforts. Hence, there is an increasing need for better monitoring to complement existing utility data: real-time, consistent, spatially-disaggregated, time-series information that can track power outages and recovery.

Satellite data of nighttime lights (NTL), can help meet this need. Unlike utility records, satellite-based measurements are collected onboard space-based platforms that can provide global and consistent coverage. Daily satellite overpasses enable repetitive measurements over time and space, and can survey, not only outages, but the speed of restoration, both within cities and in remote and isolated areas that may be difficult to reach.

While there have been many studies that have used NTL to detect blackouts [17–20], and even one on power outages from Hurricane Maria specifically [21], these studies have focused on mapping the spatial extent of the area affected by the initial power outage. Few have quantified the magnitude of electricity disruptions within a neighborhood (e.g. the % of residents who have lost electricity), nor the long-term progressive recovery after the initial event at multiple points in time. In the past, quantitatively tracking power recovery has been hindered by a lack of calibrated and scientifically validated NTL at sufficient spatial and temporal granularity. Another major limitation has been the presence of extraneous sources of noise that can overwhelm electrical lighting signals. These include stray light from the moon, airglow, and any other atmospheric constituents (e.g., dust, haze, and thin clouds) [22–25], as well as seasonal variations associated with surface-based features, such as snow, fire, and vegetation cover [26, 27], that make NTL fluctuate across time. Data that were contaminated by these artifacts (more than half of the measurements annually) had to be discarded, creating large temporal gaps in the time-series archive. Recent advances in algorithms that remove these artifacts have enabled the production of new globally-available, science-quality, daily NTL data, called the NASA’s Black Marble product (VNP46) [22].

Here, we apply Black Marble data to a disaster context, constructing high-resolution maps of electrical grid restoration at sub-neighborhood scales for Puerto Rico following the landfall of Hurricane Maria. The study measures changes in power access for various pre- and post-disaster stages during a 6-month period following Hurricane Maria in 2017 for the 8 districts, 78 municipalities (S1 Fig), and 900 administrative units (hereby termed ‘barrios’) within Puerto Rico. We rely on the synergistic use of daytime and nighttime optical satellite remote sensing [28], to downscale NTL to a high spatial resolution. The high spatial resolution allows us to examine the structural and demographic attributes of neighborhoods longest affected by power outages, and to understand how the recovery may have mitigated or exacerbated existing social vulnerabilities to hurricane impacts. Results are validated against electric utility and ground survey data to confirm the consistency of satellite observations of electricity access in both distribution and timing with known ground-based records.

## Materials and methods

Our analysis consists of four main steps. First, we use 500m daily estimates of NTL derived from NASA’s Black Marble product suite (Collection 1.1, VNP46, data access: https://blackmarble.gsfc.nasa.gov) to generate the NTL time series after Hurricane Maria. We use 195 days of NTL data spanning from September, 20 2017 (the date of landfall) to March 20, 2018. The Black Marble Product was validated over Puerto Rico before Hurricane Maria, and determined to be of sufficient quality to detect power outages and recovery time. Second, to enable detection of sub-neighborhood scale urban-lit structures, NASA’s Black Marble High Definition (HD) product (30-meter spatial resolution) is generated by downscaling the 500m nighttime satellite observations with urban built-up features extracted from Landsat-8 and Sentinel-2 satellite data and other ancillary datasets. Third, various metrics related to the spatial extent, duration, and overall impact of power outages are calculated from these satellite-derived products. The final step in the analysis is to link power recovery rates with locational, demographic, and structural characteristics within and across Puerto Rico’s municipalities. Each of these steps is described in detail in the sections below.

### Generating and validating NASA’s Black Marble standard product

NASA’s Black Marble standard product suite combines cloud-free, atmospheric-, terrain-, vegetation-, snow-, lunar-, and stray light-corrected nighttime VIIRS Day/Night Band (DNB) radiances, with daytime DNB surface reflectance, bidirectional reflectance distribution function (BRDF)/albedo, and lunar irradiance values, to minimize the influence of extraneous artifacts in the final NTL values. This includes consideration of moon-sensor geometry, surface albedo coupling, and atmospheric effects [22]. The removal of these artifacts significantly improves retrieval quality [22, 29, 30]. Atmospheric correction is based on accurate vector radiative transfer modeling of the coupled atmosphere-surface system, which helps compensate for aerosols, water vapor, and ozone impacts on the NTL radiances [31]. The lunar contribution is estimated by integrating a post-launch lunar irradiance model of the VIIRS DNB with surface BRDF/albedo retrievals [30, 32, 33]. Removal of lunar cycle radiance enables routine production of daily nighttime lights, a key requirement for disaster response and other low latency applications [27].

Validation activities in 2017 (8 months prior to the passing of Hurricane Maria) were conducted across Puerto Rico to assess the accuracy of NASA’s Black Marble product suite. This included multiple successful deployments of stable light point sources to quantify errors based on aerosol contamination and other extraneous factors. Results indicate a sensitivity enhancement of 16.95% (or 4.3 nW/cm^-2^/sr^-1^) relative to the current baseline, demonstrating the utility of the Black Marble product in detecting low-lit sources of artificial lights at night [22]. Additional assessments were conducted to establish the stability of the VIIRS nighttime cloud mask. Results from a series of benchmark tests conducted on a 5-year time series of observations across Puerto Rico (n = 1,825, Tile h11V07) indicate high probabilities of correct typing (PCT) both under moon-free (PCT = 92.66%) and moon-lit conditions (PCT = 82.06%) [22]. By minimizing data gaps, and the influence of extraneous sources of nighttime lights, the current Black Marble (Collection V001) product quality is sufficient to quantitatively detect power outages and estimate recovery times.

### NASA’s Black Marble high definition (HD) product

Black Marble NTL data provides information about infrastructure use and access patterns, but have thus far been generated at coarse spatial resolution (> 500-meter). In contrast, daytime observations based on moderate resolution optical imagery (i.e., Landsat 8 and Sentinel-2 at ~10–30 meter) provide details about the layout of the built environment [34] but are limited in their ability to distinguish electricity infrastructure and its operational status. Integration of these different data sources can improve monitoring of infrastructure dynamics at street and block scales [35], the scales needed for disaster response.

**Fig 1:**
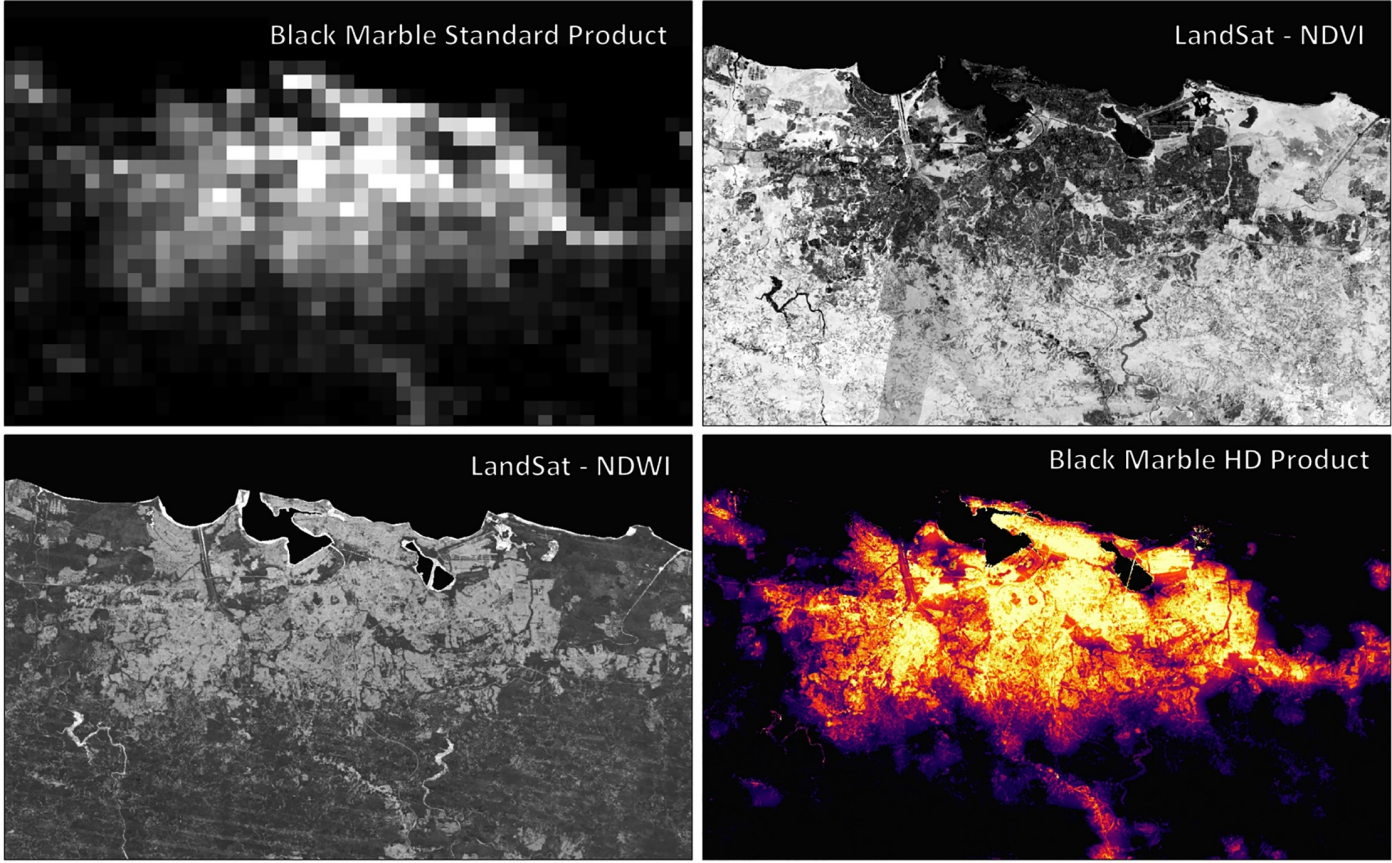
Data inputs for the production of the Black Marble HD Product to measure Puerto Rico neighborhood level electricity access. (Top right) 500m Black Marble VNP46 product (Top left) Landsat 8/Sentinel 2 NDVI (Bottom left) Landsat 8/ Sentinel 2 NDWI (Bottom right) Black Marble HD 30m Product.

We call the result of this integration effort the Black Marble HD product, a fine-resolution (30m) version of the standard 500m Black Marble Product. Black Marble HD is based on 30 meter retrievals of the Normalized Difference Urban Index (NDUI) [28]:

$$NDUI=\frac{{NTL}_{n}-NDVI}{{NTL}_{n}+NDVI}$$ (1)

where *NTL*_*n*_ is normalized Nighttime Light (NTL) from the 500m Black Marble product (VNP46) (Fig 1, top left) calculated as:

$${NTL}_{n}=\frac{NTL}{{NTL}_{max}}$$ (2)

For this study, we used a NTL_max_, equal to 180 nW·cm^-2^·sr^-1^, because it was the maximum radiance value over Puerto Rico. Thirty-meter spatial resolution NTL data are then generated by resampling the 500m NTL data using standard cubic convolution interpolation methods [36]. NDVI (in Eq 1) is Normalized Difference Vegetation Index [37]:

$$NDVI=\frac{\rho_{NIR}-\rho_{NIR}}{\rho_{NIR}+\rho_{red}}$$ (3)

Where *ρ_NIR_* and *ρ_red_* are the surface reflectance of NIR and red bands respectively. The 30-meter NDVI was calculated from a high frequency period of combined Landsat-8 and Sentinel-2 (~2–3 days) surface reflectance products [38] (Fig 1). When a Landsat-8/Sentinel-2 scene is missing or cloud-contaminated, the surface reflectance retrieval is gap-filled from the nearest weekly or monthly values, following the same algorithm compositing process as established by the Web-Enabled Landsat Data (WELD) project [39]. Additional water features, based on the Normalized Difference Water Index (NDWI) (Fig 1), are calculated from HLS surface reflectance data [40], and are used to screen out water-contaminated areas that may be confused as urban-built up pixels:

$$NDWI=\frac{\rho_{green}-\rho_{NIR}}{\rho_{green}+\rho_{NIR}}$$ (4)

where *ρ_green_* and *ρ_NIR_* are the surface reflectance of green and NIR bands respectively. The threshold of 0 for NDWI was used to identify a pure water surface.

For visualization purposes, NDUI values are enhanced along urban roads and highways, using GIS data from Open Street Map (OSM). OSM is a collaborative project that provides geospatial datasets created using a combination of aerial photographs, portable GPS devices, and other open access data sources with high accuracy [41–45]. The OSM road network is extracted and rasterized to match the NDUI layer, and NDUI values that lie on roads are enhanced by 120% to make them legible. The enhancement is applied for all days, so it does not affect the time series information (days without power), only the allocation of power outages to streets.

A final Black Marble HD image for the island of Puerto Rico is shown in Fig 1, and a sample of municipality-level Black Marble HD images produced at different stages of recovery are shown in S3 Fig. We note that the Black Marble HD retrievals are designed to monitor sub-neighborhood scale variations in NTL features. As such, they should not be used to quantify outage conditions for individual buildings.

### Power outage metrics

Statistics on the duration of outages were calculated across multiple spatial and temporal scales. We devised three metrics to quantify levels of energy service provision and access: (1) relative recovery in NTL (% Recovery), (2) net number of days without electricity (NDWE), and (3) net total customer hours of interruption (CHI).

The relative recovery in NTL (Eq 5) was calculated for each disaster stage *i*:

Where *NTL*_*i*_ is NTL radiance at stage *i*:

$$\%Recovery=\frac{{NTL}_{i}}{{NTL}_{0}}$$ (5)

Where *NTL*_*i*_ is NTL radiance at stage *i*, *NTL*_*0*_ is the pre-hurricane NTL radiance.

Finally, the net number of days without electricity (NDWE) and net total customer hours of interruption (CHI) are defined as:

$$NDWE=\sum_{i=1}^{3}(1-\frac{{NTL}_{i}}{{NTL}_{0}})*D_{i}$$ (6)

$$CHI=\sum_{i=1}^{3}(1-\frac{{NTL}_{i}}{{NTL}_{0}})*H*24*D_{i}$$ (7)

*H* is the estimated number of households from the US Census. *D*_*i*_ is the number of days for stage *i (60 days on average*). Each metric was estimated using the native Black Marble grid scale (500-meter), and then aggregated at regional (senatorial district), municipality and barrio levels for three key disaster stages.

The above-mentioned metrics should only be estimated after properly accounting for systematic sources of uncertainty and measurement error in the VIIRS Day/Night Band time series. Since the accuracy of the VIIRS cloud mask has been shown to affect detection of outage conditions [30,46], multi-date aggregation periods (n = 4 days) were applied to the daily Black Marble NTL product to reduce errors of omission. In addition, it is important to properly correct for atmospheric- and lunar BRDF effects, as these can considerably alter daily NTL radiances (S4 Fig). Additional sources of measurement error (e.g., seasonal vegetation effects and aerosol contamination) can also lead to incorrect estimations of radiances, and thus incorrect estimates of timing and duration of outages.

### Linking power outage metrics to community characteristics

We used ancillary census survey data from the American community survey (ACS) [47–49] to examine how the impacts of Hurricane Maria were distributed (who was most affected, what types of households). The ACS has block group level counts for a variety of demographic and housing characteristics. Three ACS variables were used: median household income in the past 12 months (B19013), households by presence of people 60 years and over (B11006), and housing units (B25001).

The proportion of elderly households within each block group was calculated as:

$$Prop\;el{derly}_{b}=\frac{E_{b}}{\sum_{i=0}^{n}{Pop}_{b}}$$ (8)

Where *b* is the blockgroup within each municipality, *E* is the number of households with residents over 60 years old (B11006) in blockgroup *b*, and *Pop*_*b*_ is total population within block group b. Housing density was calculated as the number of housing units in a blockgroup (B25001) divided by the total area of land within each blockgroup.

To examine regional differences in recovery, we used a remoteness index (S2 Fig), that was adopted from Kishore et al. (2018), which is defined according to the travel time to urban centers with total populations of at least 50,000 persons [4]. Based on this remoteness index, Puerto Rico’s barrios are grouped into eight categories according to percentile from least remote (category 1) to most remote (category 8).

## Results

### Validation of satellite-based outage conditions

**Fig 2:**
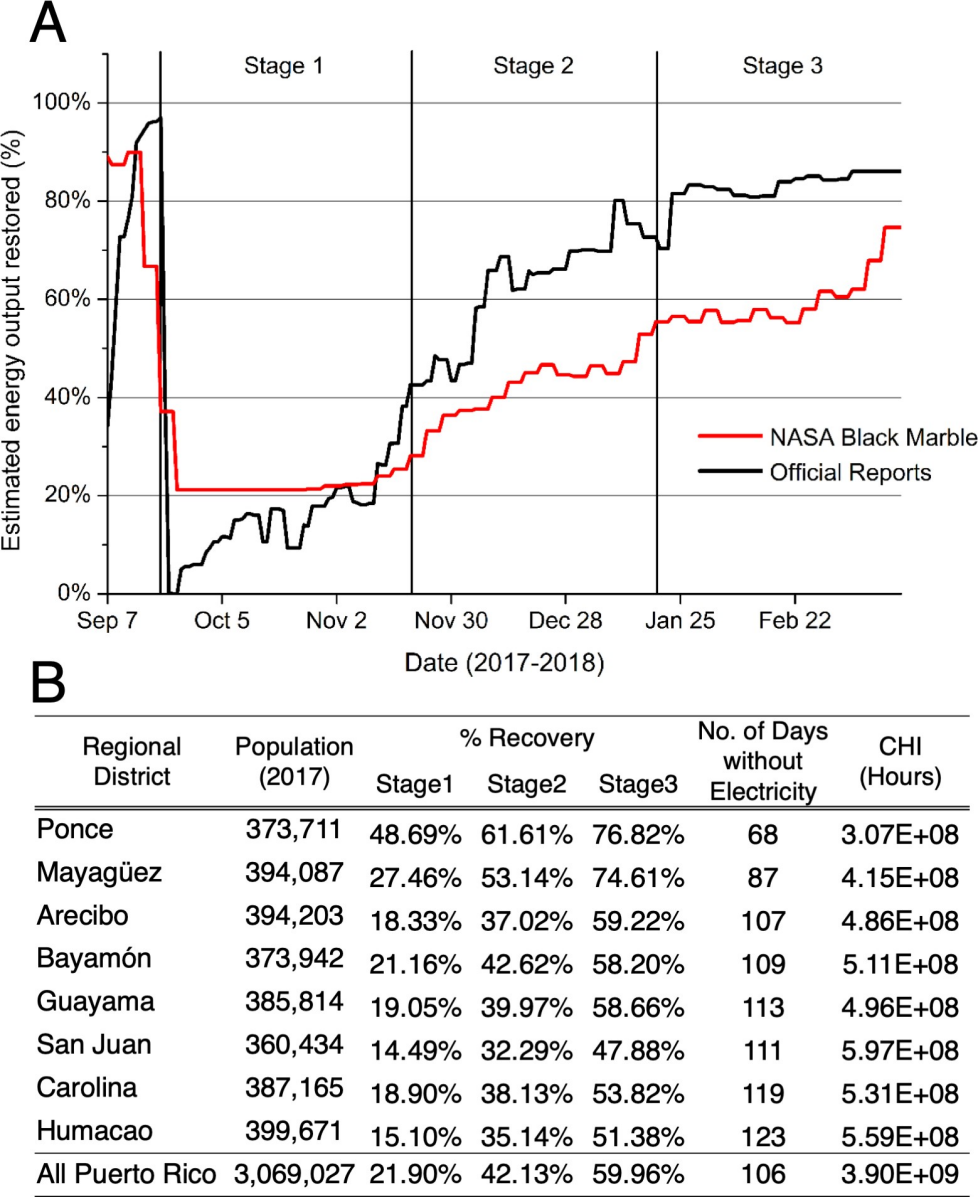
Satellite-derived nighttime lights products agree with official records of total electricity generation following Hurricane Maria in Puerto Rico (R = 0.839, SE = 0.1601, P < 0.0001, n = 195 days). (A) Comparisons between total shares of peak load restored and lighting patterns Results show distinct variations between three disaster stages. (B) District-specific outage metrics include ‘% Recovery’ (scaled units of radiance relative to pre-event values), number of days without electricity (NDWE), and customer hours of interruption (CHI) (see Supplementary Information for details).

During the aftermath of Hurricane Maria, Puerto Rico’s Electric Power Authority (PREPA) served as the primary data source for tracking power failures in Puerto Rico. Following the reestablishment of PREPA’s ground monitoring networks, self-reporting of island-wide electricity reconstruction efforts became routinely available. These reports estimated total rates of grid connected and temporary power on a weekly basis, aggregated at the island level [50]. We compare these reports to our NTL-derived measurements of outage conditions for each of Puerto Rico’s eight regional districts (Fig 2).

The NTL approach tracks the timing of electricity recovery reported by PREPA, showing average differences of 17.9% across the period of evaluation. Three distinct post-disaster stages can be identified in both the satellite and utility measurements: Stage 1, the immediate damage assessment period following Hurricane Maria (Sep. 20 to Nov. 20, 2017); Stage 2, the ensuing relief efforts led by FEMA and the US military (Nov. 21, 2017 to Jan. 20 2018); Stage 3, the recovery efforts led by PREPA and the US Army Corps of Engineers (Jan. 21 to Mar. 20, 2018).

Over the assessment period, we estimated a total net loss of 3.9 billion customer hours of electricity service (Fig 2). While these estimates compare well with PREPA’s independent assessments (see Fig 2), there are several situations where we would expect for the two estimates to diverge. One instance is if grid-connected electricity or generator access were provided during the day, but not at night (e.g., as a result of rolling brownouts). Hence, in some areas, NTL-based estimates may not capture short-term availability of electricity well. In addition, the most significant differences between the two measurements occur at the end of Stage 2 and beginning of Stage 3. This divergence is expected since the NTL-based estimates are capturing different electricity end uses than official reports. The restoration of street lighting, which accounts for a large portion of the satellite-derived signal, often lags behind the restoration of essential energy infrastructure (e.g., schools and hospitals), as well as commercial electricity. This, in effect, creates a temporal bias in the satellite-derived products (particularly during Stages 2 and 3) relative to official reports.

### Patterns in Puerto Rico’s outage losses and recovery

**Fig 3:**
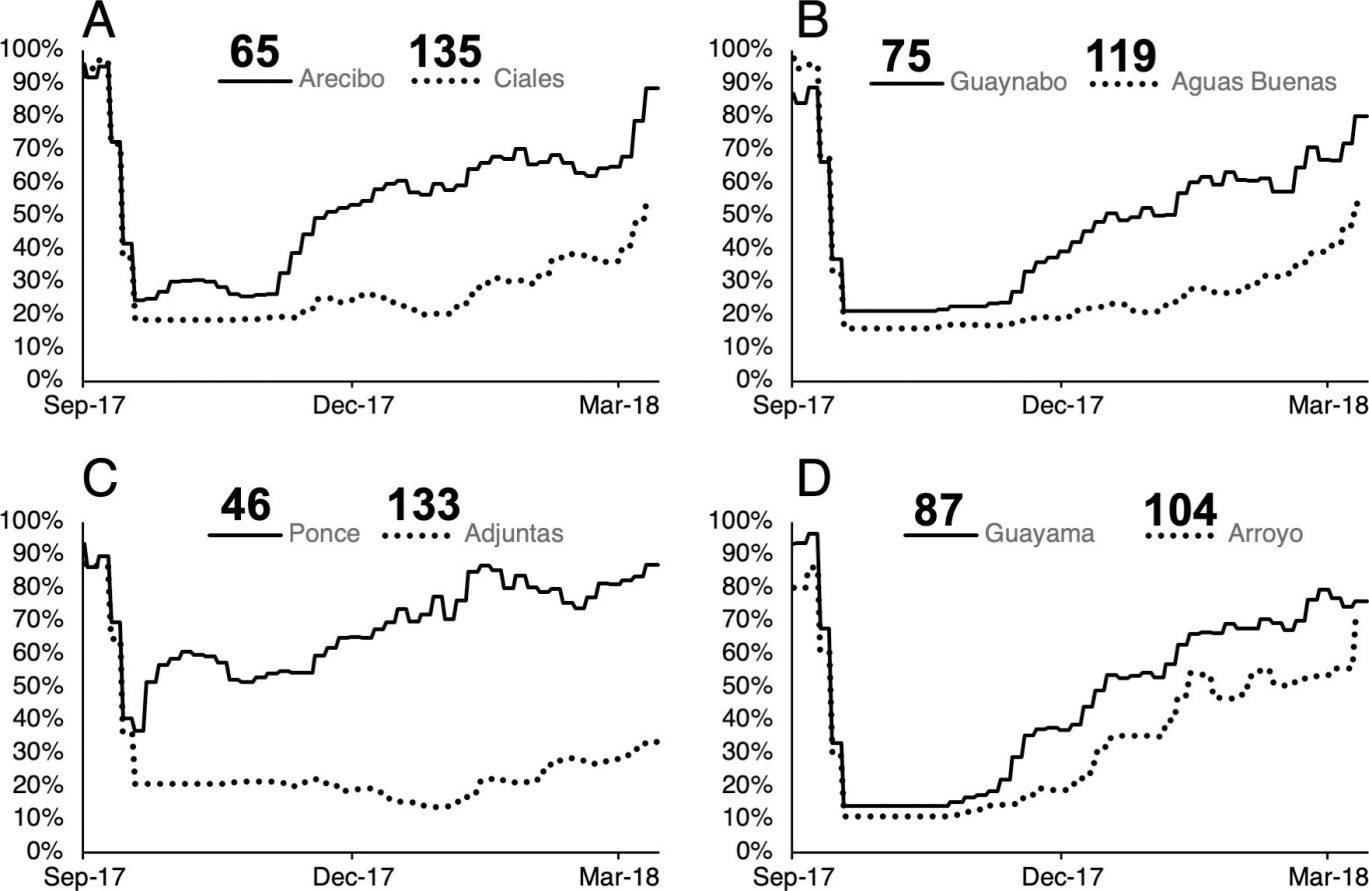
Differences in recovery time across Puerto Rico’s rural municipalities. Daily time series profiles (%Recovery) (Detailed Methods in Supplementary Information, Satellite-based retrieval of power outage metrics) show significant variation in outdoor lighting conditions between the regional urban centers and affluent suburbs of (A) Arecibo, (B) Guaynabo, (C) Ponce, and (D) Guayama (shown in solid lines), and the adjacent rural and peri-urban towns of (A) Ciales, (B) Aguas Buenas, (C) Adjuntas, and (D) Arroyo (shown in dotted lines). Individual NDWE results (numbers on top of legend) correspond to total cumulative levels of energy access (i.e., available on- and off-grid electricity) for each municipality.

Tracking electricity recovery at the regional level highlights factors that are known to influence recovery rates: storm exposure, infrastructure quality, proximity to power stations, and remoteness from large urban areas. Puerto Rico’s southwest region (i.e., Ponce and Mayagüez, districts which sustained lower peak wind gusts (< 95 mph) experienced comparatively minor disruptions in electricity service (average number of days without electricity [NDWE] < 88 days; customer hours of interruption [CHI] < 420 million hours, (Fig 2)). The Ponce district benefited from recent upgrades to its distribution system, as well as the addition of backup electricity supply. In addition to the lower initial exposure, these infrastructure investments helped the district of Ponce to recover to 60% recovery within days of Maria’s passing (Fig 3).

In contrast, the districts of Arecibo, Bayamón, and Guayama which were harder hit by the storm (sustaining peak wind gusts > 95 mph) experienced slower rates of recovery, leading to longer interruption periods (NDWE > 105 days; CHI < 520 million hours) (Fig 2). These districts did not reach 60% recovery until the end of December, or later—three months after the initial storm event (Fig 3).

Slow rates of recovery in electricity service were also observed across the north and eastern districts of San Juan, Humacao, and Carolina (NDWE > 110 days; CHI > 530 million hours) (Fig 2). During the aftermath of Hurricane Maria, officials made it a priority to reestablish service to the San Juan Metropolitan region. However, since the nearby Palo Seco central power plant (602 MW) was tentatively taken offline (due to sustained damages), and the bulk of the remaining electric generation capacity (3,565 MW) was located in the southern portion of the island, reestablishing service in the capitol required the repair of long-distance transmission lines that could bring power from the south to the north. These circumstances resulted in outages across Puerto Rico’s north and eastern districts through December, 2017 [51].

In addition to regional differences in districts, there were gaps in electricity restoration between urban and rural municipalities within the same district. Despite similar geographic characteristics and levels of storm exposure, most urban municipalities experienced significantly faster recovery times compared to their neighboring rural municipalities (Fig 3). On the whole, rural municipalities in Puerto Rico had an average NDWE of 131 days, compared to 115 days for their urban counterparts (which included surrounding suburbs). Quicker recovery rates in urban municipalities are consistent with standard restoration protocols that aim to restore electricity to the largest number of customers in the least amount of time [52]. Protocols therefore favor the allocation of electricity to population dense areas, to benefit the most people.

**Fig 4:**
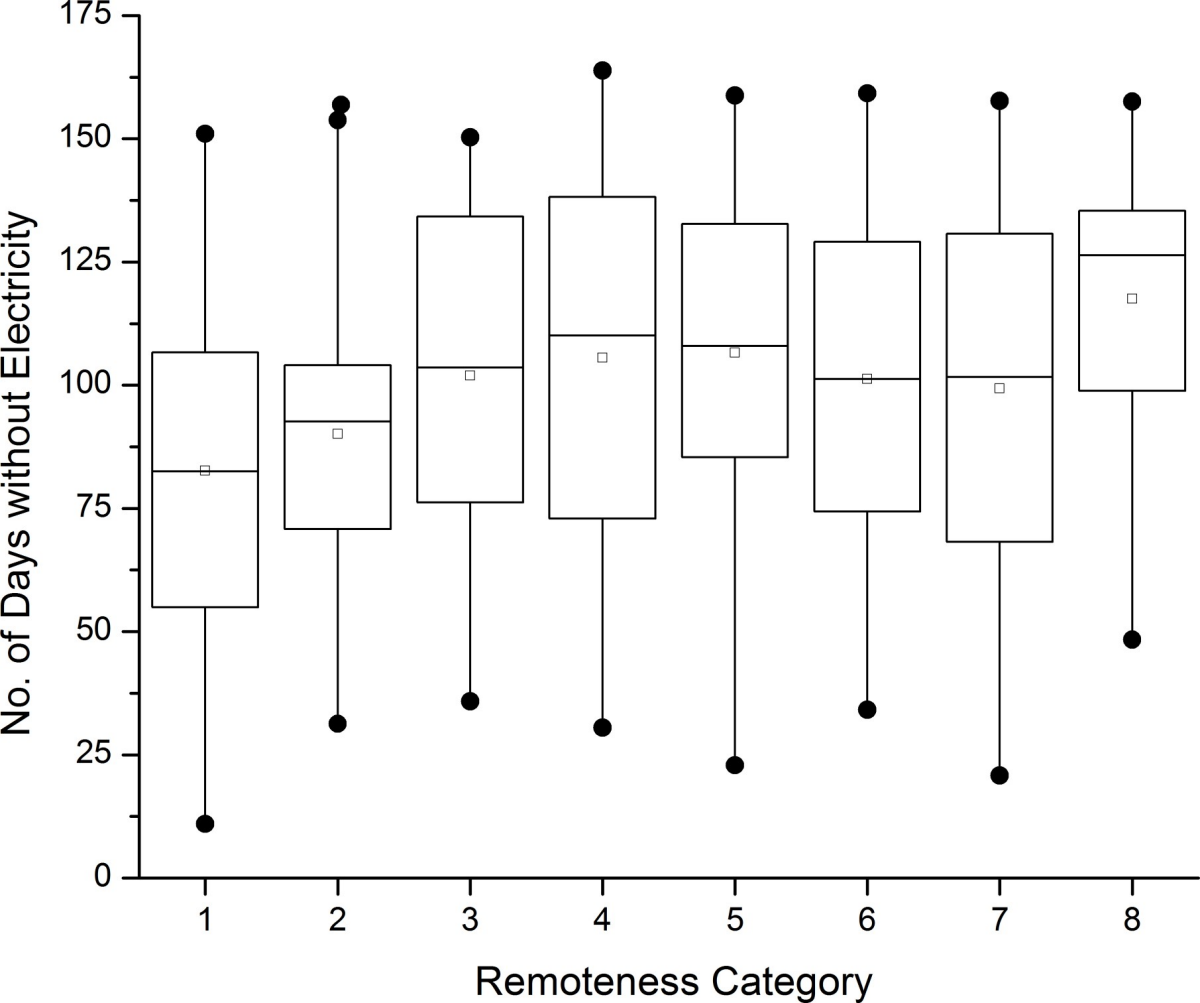
Distribution of NDWE according to remoteness category. Box plots show the medians (dark bars) of recovery time for barrios in different remoteness categories. Recovery time progressively increases as remoteness increases (from category 1, least remote, to 8, most remote. Boxes indicate the interquartile range (25/50/75 percentiles); vertical lines indicate the min/max range, and points denote outliers.

In addition, rural areas are often more difficult to access because of narrower or lower quality roads that are easily blocked by storm debris and obstructions. Barrios that were more remote (i.e. took longer to reach from urban areas) were among the last to get power restored (Fig 4). For example, the western interior of the island (e.g. Adjuntas and Ciales districts), is both remote and mountainous and therefore was affected by cascading post-storm hazards like landslides. Previous analyses have corroborated the association between remoteness and the severity of disruptions in electricity services using ground surveys [4].

**Fig 5:**
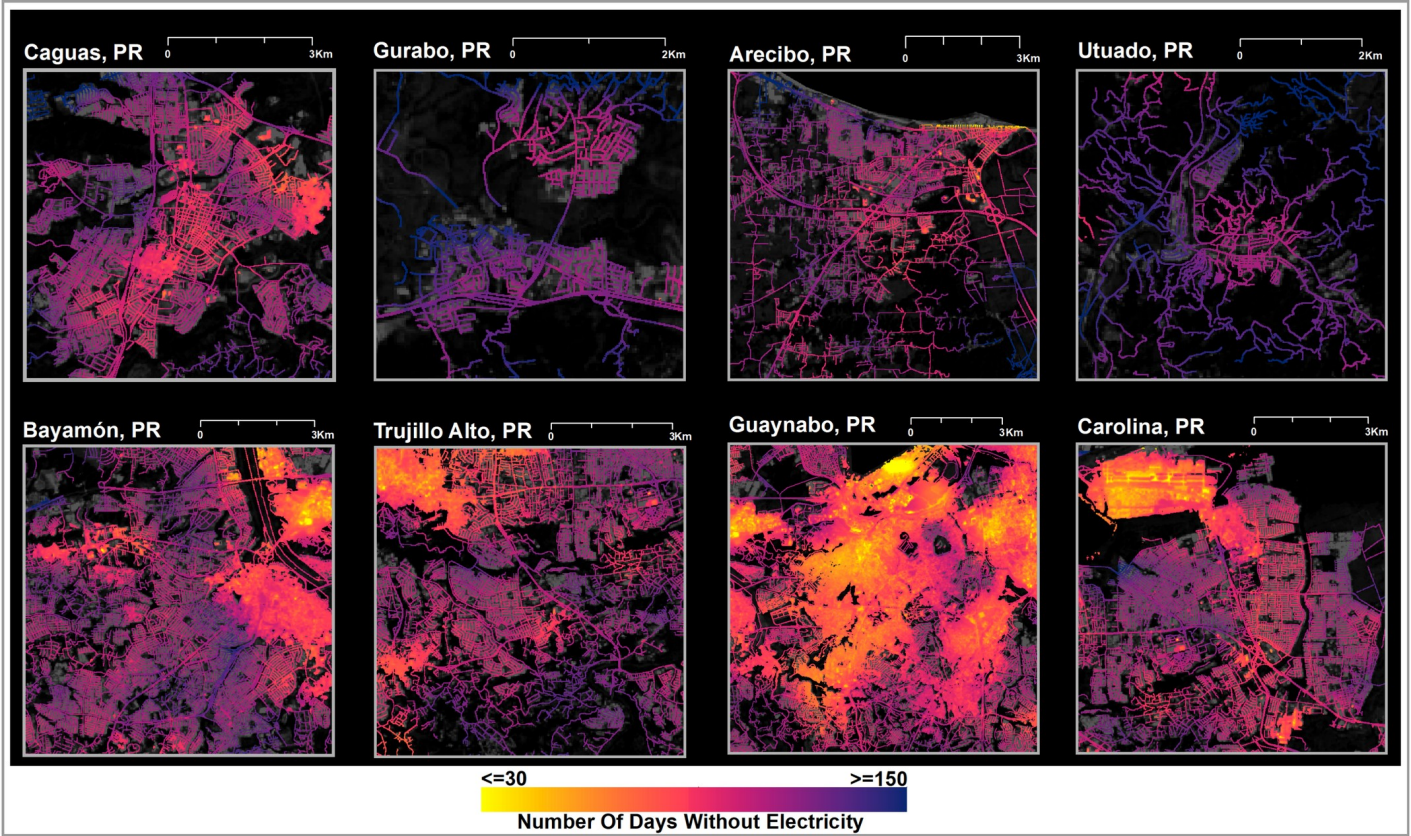
Progression of electricity recovery across a subset of Puerto Rico’s neighborhoods using NASA Black Marble high definition nighttime lights. (Top) Deficits in energy access were found between regional urban centers (e.g., Caguas and Arecibo) and adjacent rural towns (Gurabo and Utuado respectively). (Bottom) Fragmented patches of recovery demonstrate the significant variations in NDWE (between September 20, 2017 and March 20, 2018) within the San Juan metropolitan region.

Despite these trends between municipalities, we expected neighborhoods within each municipality to have similar rates of electricity recovery, given their spatial proximity and equivalent remoteness. However, the analysis identifies large discrepancies in recovery times (sometimes >60 days) between different neighborhoods, and even parcels, within Puerto Rico’s municipalities (Fig 5). Amongst San Juan’s census block groups, 4% fully recovered to pre-Maria electricity levels in less than 60 days (usually public or governmental land uses), 40% in 60–90 days, and 53% in 90 to 120 days. Three percent of San Juan’s block groups still hadn’t recovered after 120 days. Despite sustained electricity restoration efforts undertaken in San Juan, power failures persisted in the neighborhoods of Barrio Israel and Barrio Oriente, in the public housing projects (‘Residenciales’) El Flamboyan, and the adjoining Campo Rico area. They also persisted in the detached homes at the urban outskirts—southern Trujillo Alto, southern Carolina, and eastern Bayamón (Fig 5).

**Fig 6:**
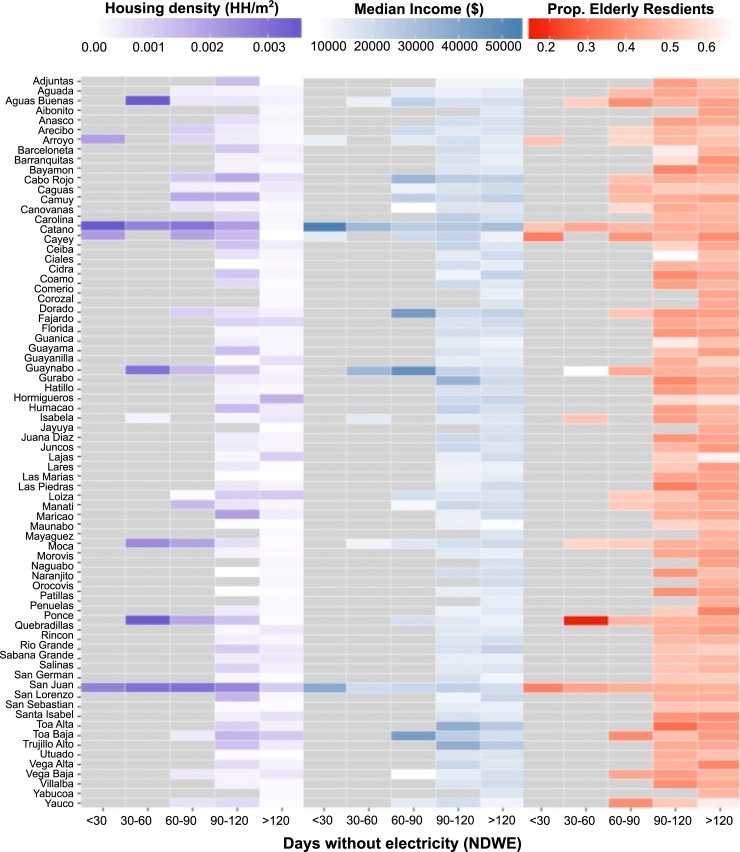
Interaction between demographic vulnerabilities and density-based power restoration protocols. (Left) Housing density (households per square-meter) by recovery rate for all of Puerto Rico’s municipalities. White indicates lower housing densities and dark purple indicates the highest housing densities. Days without electricity (NDWE) consistently increases with declining housing densities across Puerto Rican municipalities. (Center) Average Median Income in USD ($). (Right) Proportion of elderly residents.

Within almost all of Puerto Rican municipalities, NDWE increased with declining housing densities, indicating that, even at a local scale, recovery efforts prioritized the number of households affected when restoring electricity (Fig 6). Following the same logic applied to urban and rural municipalities, detached homes and lower-density neighborhoods within Puerto Rican municipalities were serviced later because the resident numbers were lower. A small minority of municipalities (7%) were exceptions to this rule. In Hormigueros, Ciales, Lajas, Quebradillas, Santa Isabel, and Vega Baja, housing density did not determine which areas of the municipality had their service restored first, though these municipalities—none of which host large urban areas—had little housing density heterogeneity within their boundaries. Within municipalities with large cities (like San Juan and Ponce), recovery disparities were greater. For example, the urban area of Ponce had an average NDWE of 96, versus 148 days for the rest of the municipality (see table in S1 Text). In contrast, municipalities with only small town centers, (e.g. Barranquitas, Comerio, Orocovis) saw no difference in urban and rural electricity recovery.

### Interactions between power restoration rate and vulnerable populations

**Fig 7:**
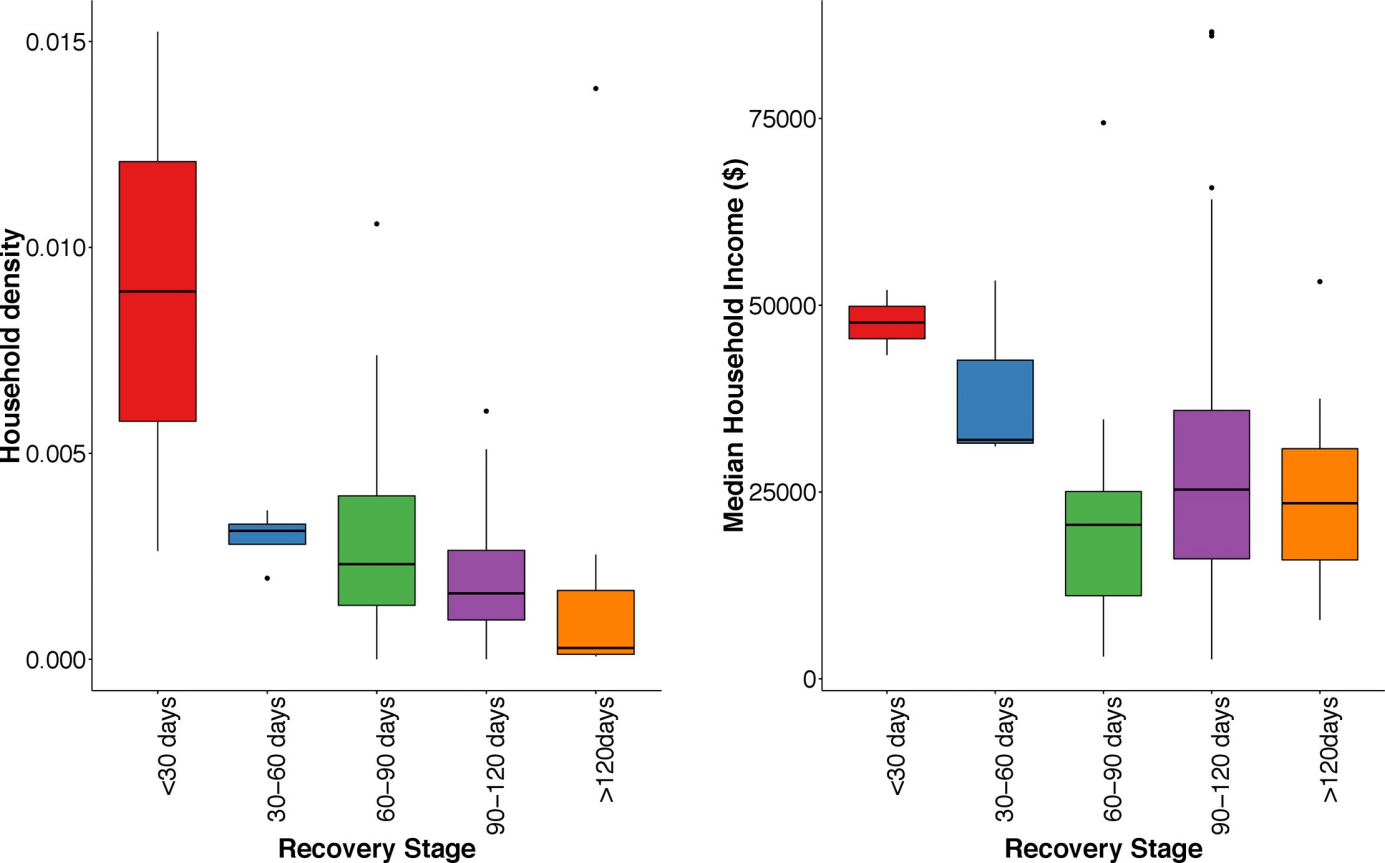
Boxplot of recovery stage estimates for different income levels and household densities within Puerto Rico’s urban municipalities (those with population> 50,000). (Left) lower density neighborhoods within Puerto Rico’s urban areas have progressively slower recovery times. (Right) Wealthy neighborhoods have quicker electricity restoration than poorer neighborhoods.

In examining who was most affected by Hurricane Maria’s long-term power outages, the results show that populations with existing vulnerabilities were most disadvantaged by density-based recovery protocols (Fig 7). Households that recovered in Stage 1 earned almost double that of households recovering in Stage 2 or 3. Disparities in recovery between households of different incomes were greatest in Puerto Rico’s large cities, with the poor shouldering much longer power outages than average households in San Juan, Bayamon, Carolina, Dorado, Ponce, Toa Alta, and Toa Baja (Fig 6). Rural municipalities saw much less variation in recovery rates, and also in disparities between the populations who benefited from them. There were occasional but notable exceptions to this general trend. Low-income populations were helped by density-based restoration protocols in the municipalities of Camuy, Luquillo, and Mayagüez, where low-income populations live closer to the urban core or in multi-family units.

Density-based recovery protocols had a less uniform impact for seniors (Fig 6). In Yauco, residential areas with fast recovery rates (less than 90 days) were home to a lower proportion of seniors (40%), compared to those with a NDWE>90 days (60%). However, that trend is reversed in Mayagüez.

## Discussion

Hurricane Maria caused over 4.0 billion customer hours of interruption–the longest recorded blackout in U.S. history, and the second largest power outage in recorded history after the Typhoon Haiyan [53]. Following the hurricane, federal agencies spent months helping repair Puerto Rico’s grid. Though PREPA, Puerto Rico’s current utility monopoly, uses an outage management system to identify customers without power, outage reports created from this data are aggregated before they are sent to public and government agencies. Prior to these results, only a coarse understanding of the Puerto Rican electricity grid recovery effort was available.

Many of the results gleaned from our finer-scale map of electric grid recovery are not surprising. Rural, remote areas, and regions most damaged by the storm were expected to recover slower, both because the complexity of restoration work to be done in these areas, and the resources necessary to do that work are greater. In responding to the storm, we found that density-based restoration protocols were consistently applied across multiple scales, prioritizing both urban municipalities over rural municipalities and dense multi-family housing structures over detached housing. For 90% of Puerto Rico’s municipalities, we found little evidence of utilities veering from this standard, logical protocol.

Though the impacts and restoration results were expected, their impact on vulnerable populations was not. An unintended consequence of the density-based restoration meant that in a majority of Puerto Rico’s municipalities, and especially in bigger cities, low-income residents were burdened with longer recovery times. Modern, middle and high-income residential developments in Puerto Rico often have one electric connection point to the entire community and underground power lines. Utilities maximize their restoration numbers by prioritizing those neighborhoods over older, poorer, low-rise sections of the city with above ground electricity infrastructure and multiple-connection points. Furthermore, more expensive or newer housing developments were often closer to the urban core, especially in Puerto Rico’s largest population centers.

This density-vulnerability tradeoff is not unique to Puerto Rican cities or Hurricane Maria, but would likely play out similarly in many parts of the world. Larger urban areas create the conditions for larger inequalities in incomes and land values, resulting in residential reshuffling. Decentralization of the poor to low density suburbs is a well-documented trend in American cities [54, 55], and increasingly across the globe [56–58], one that continues to escalate with urbanization. Due to the desirability of accessible jobs, services, and public amenities in large urban areas, land prices near high-density central business districts increase, making rents unaffordable for the urban poor [59]. As a result, in many cities, a density-based protocol for power restoration would disadvantage low-income populations.

This might not matter when outages are brief, but in long-term outages, the risk of irreversible morbidity and mortality impacts in vulnerable populations (the elderly, poor, and disabled) is much higher than for average households. Many studies have established the link between socioeconomic factors and direct and indirect fatalities from disasters [60–62]. In conjunction with this literature, the observation that restoration protocols may unintentionally, but systematically, cause vulnerable populations to wait longer before gaining electricity access is a significant finding. Our results add to the growing literature on interactions between three dominant trends of the 21st century—increasingly large and frequent disasters with long-duration impacts, urbanization, and decentralization of the urban poor. The density-vulnerability tradeoff underscores a previously unnoted second-order effect in how the processes that shape urbanization, and who lives where, can in turn shape disparities in communities’ resilience to disasters.

A second and more direct result of this study is the development of the approach itself. We demonstrate that satellite data can be used to reliably and accurately map real-time electrical grid restoration at fine spatial scales. The approach developed is useful for many different actors in the emergency response community, because it addresses information gaps that utility data cannot fill due to how utilities detect power outages, how outage data is shared, and who utilities are interested in monitoring.

For utilities themselves, NTL-based approaches are useful because they are based on data that is collected automatically. In contrast, real-time information on power outages and recovery is often not collected by utilities in an automated way. Historically, power companies have relied on supervisory control and data acquisition systems (SCADA) to detect problems in higher tiers of the transmission system, like sub-stations. However, since distribution lines are unmonitored, this data does not describe the extent of damage or when power is restored. Instead, localized conditions are captured by crews that visually assess the damage and receive customer calls [63], a costly and time-intensive endeavor when the extent of damages is immense. Furthermore, the manual assessment used by utilities are less effective when outages occur at night and customers are not awake, or after severe disasters when downed or electricity-reliant communication infrastructure can make call-based reporting infeasible [64]. In these situations, the NTL-based approach can provide an immediate first assessment to help utilities know the areas that need to be scoped. Some utilities are responding to the lack of automated data by installing smart meters to automatically receive household level information on power outages, but currently there are large gaps in deployment. Smart meters cover around 50% of US consumers [65], and are lagging behind in many developing countries [66] where technical and budgetary constraints are greater. Since NTL data is available, globally, NTL-based outage information can help bridge the gap, while the deployment of smart meters expands.

For emergency management professionals and insurance companies, NTL-based approaches are useful because, unlike utility data, they are consistently measured, comprehensively collected, and publicly-available across impacted regions. In the US, once utilities collect power outage data, only major electricity providers are required to report it to the public, most often via the utility’s website. Small producers and municipal power companies often have no website or reporting protocols. Even when data is released on utility websites, it is rarely actively reported to data custodians outside of the local service area [11]. Instead, outage data is scraped from utility company websites (as in the Department of Energy’s Environment for Analysis of Geo-Located Energy Information (EAGLE-I)) [67], and then spatially aggregated to county or zipcode levels to make it more consistent [68, 69], which diminishes its value as actionable information. Currently there is no centralized map that displays outages down to the street level [13].

Creating a centralized map from utility data is an enormous undertaking, as there are almost 2,000 utilities involved in owning and operating the US generation, transmission, and distribution infrastructure—each with their own disjoint reporting protocols [12]. The lack of consistent and comprehensive data across the US’s piecemeal electrical grid makes it difficult to coordinate a response amongst emergency managers, insurers, governments, and the public, acting at different scales, especially in region-wide outage events like hurricanes and storms. Furthermore, no utility releases detailed historical power outage information for post-event assessments—when and where they occurred, how long they lasted, and how many people lost power. This data is imperative for learning from past events to improve disaster response, and to identify vulnerabilities in the power supply system.

Historical outage data is useful for international benchmarking as well. For example, one key component of the United Nations Sendai Framework for Disaster Risk Reduction—an agreement between 187 countries aiming to reduce disaster risks and losses—is a monitoring and reporting process. This process consists of 38 indicators meant to track global progress towards substantially reducing disaster risks and losses [70]. Currently, the International Disaster Risk Reduction Working Group (IDRR) has concluded the US does not have the ability to respond to many of these indicators, one of which is indicator D-5, an estimate of the number of disruptions to basic services attributed to disasters (White House Sub-committee on Disaster, Sendai Framework Reporting SDR Brief, personal communication, 11/4/18). Since NTL satellite data is already continuously archived, the NTL-based approach addresses this gap, and the power outage products are producible beyond the US, in countries without utility data reporting or collection systems in place.

Finally, NTL-based approaches are a useful counterpart to utility data for disaster response efforts and vulnerability assessments because of what is measured. Even when outage and recovery information is automatically detected and publicly shared, utilities only monitor their customer’s power data—i.e. the purchasers of electricity generated at the power plant. They do not capture power availability in areas with decentralized electricity systems (e.g., solar, gas, and battery-powered distributed generation and microgrids), which are increasingly common in remote areas. In decentralized systems, responsibility for operations shifts towards the end-user, so utilities have no information on the state of the infrastructure involved [71], or whether populations that use these systems have access to electricity [72]. Since most disaster response actors are interested in the entire population, the exclusion of decentralized end-users is another data gap in understanding power outages and recovery, and an important one in the least developed countries where distributed generation may play a larger role [73]. The NTL-based approach does not differentiate between centralized electricity generation and decentralized electricity generation.

However, there are also limitations to using satellite NTL data to monitor power restoration. NTL imagery primarily captures outdoor street lighting, so associations with residential customers that have lost power are not direct. Furthermore, though the standard Black Marble product is available at moderate resolution (500 m) and is further downscaled here, it is still impossible to disaggregate the different lighting end uses captured and attribute losses or recovery to specific customers. Instead, survey data from the census is used to estimate how many people, not customers, live in an affected area. Hence, this method is not a replacement for utility reporting altogether, and may be subject to estimation errors brought on by faulty population accounting in countries with poor statistical collection systems.

In summary, the results demonstrate that near-real time satellite products, like the Black Marble, can provide the kind of evidence-based information required to inform disaster response strategies, characterizing the magnitude and duration of large-area power outages, and linking to fine-scale demographic data about who is affected. This case study is a first step in a continuous, real-time, global monitoring effort. Future work incorporating these data products into disaster response communities has the potential to better address the needs of vulnerable populations and save lives.

## Supporting information

S1 Text Description of data availability and table of municipality level urban vs. rural recovery times. (DOCX)

S1 Fig A map of municipalities on Puerto Rico’s main island. While Hurricane Maria weakened slightly before making landfall on September 20 in southeastern Puerto Rico, the storm’s track (shown in S2 Fig) was a near worst-case scenario. It ripped directly across the island with sustained winds of 155 miles per hour (250 kilometers per hour). All of the municipalities in Puerto Rico saw outages after the storm.
(TIFF)

S2 Fig Ancillary layer describing the population of Puerto Rico’s municipalities, location of power stations, and their remoteness from large urban areas. (A) Population count estimates derived from Gridded Population of the World (GPW) collection 4 products. The National Hurricane Center (NHC) track of Hurricane Maria is shown as a purple line. (B) Heatmap of average travel time to population centers of at least 50,000 individuals using local road networks across Puerto Rico–adapted from Kishore et al. (2018) (4).
(TIFF)

S3 Fig Electricity recovery across Puerto Rico’s municipalities. NASA Black Marble high definition nighttime lights subsets show recovery of electricity before Maria and at each state of recovery in, e.g. (A) Caguas (Population = 134,481) and (B) Arecibo (Population = 94,658), and adjacent rural towns e.g., (C) Gurabo (Population = 46,406) and (D) Utuado (Population = 32,776).
(TIFF)

S4 Fig Time series plots illustrate pixel-based (500-meter) nighttime lights patterns across two residential communities in Puerto Rico. The standard 500-meter Black Marble product (VNP46) is shown to reduce noise, particularly by removing artifacts stemming for lunar reflectance anisotropy effects. The original at-sensor cloud-corrected VIIRS Day/Night Band radiances are shown as red lines (units of nW·cm^-2^·sr^-1^). Those resulting from NASA’s Black Marble nighttime lights product are shown as blue lines. The vertical lines correspond to the passing of Hurricane Irma and Maria, respectively. The trailing dotted black line tracks the lunar phase.
(TIFF)
