# Supplementary material for: Satellite-based assessment of electricity restoration efforts in Puerto Rico after Hurricane Maria
Source: PLoS One. 2019 Jun 28;14(6):e0218883. doi: 10.1371/journal.pone.0218883 (PMC6599127; doi:10.1371/journal.pone.0218883)
Supplement: S1 Text — (DOCX) [file pone.0218883.s001.docx]

**Supporting information**

**S1 Text. Description of data availability**

NASA Black Marble HD imagery data prepared for this paper can be obtained from the NASA’s Disasters Data Portal (<https://disasters.nasa.gov/hurricane-maria-2017/>). NASA Black Marble products are being made available both retrospectively, via NASA’s Level 1 and Atmosphere Archive and Distribution System (<https://ladsweb.modaps.eosdis.nasa.gov/>), and in forward (near-real time) data streams, via NASA’s LANCE data system (Land, Atmosphere Near Real-time Capability for EOS) (<https://earthdata.nasa.gov/earth-observation-data/near-real-time>).

**S1 Table. Municipality level Urban vs. Rural recovery times**

| Name | **Urban Population** | **Urban Average Person Recovery (days)** | **Rural Population** | **Rural Average Person Recovery**  **(days)** | **Rural/Urban Recovery Rate Difference**  **(days)** |
| --- | --- | --- | --- | --- | --- |
| Adjuntas | 5042 | 134.26936 | 13305 | 148.1717241 | 13.90236411 |
| Aguada | 36068 | 115.2456253 | 6138 | 118.0517034 | 2.80607814 |
| Aguadilla | 47583 | 103.3194698 | 10686 | 109.5890191 | 6.269549246 |
| Aguas Buenas | 16869 | 142.0751983 | 12245 | 146.9951784 | 4.919980094 |
| Aibonito | 16203 | 130.5689567 | 8144 | 129.3154431 | -1.253513568 |
| Anasco | 17179 | 108.2040073 | 12901 | 128.7421534 | 20.53814616 |
| Arecibo | 56472 | 113.5113043 | 38116 | 113.8348794 | 0.323575114 |
| Arroyo | 17756 | 132.5606539 | 2665 | 133.6385707 | 1.077916763 |
| Barceloneta | 18042 | 132.1960576 | 7355 | 128.8621464 | -3.333911191 |
| Barranquitas | 17404 | 141.8339042 | 12731 | 141.4588929 | -0.375011252 |
| Bayamon | 193212 | 110.7277222 | 4679 | 147.9053279 | 37.17760576 |
| Cabo Rojo | 32673 | 109.5286045 | 18304 | 111.606998 | 2.078393594 |
| Caguas | 119501 | 118.1333466 | 21639 | 139.8689749 | 21.73562833 |
| Camuy | 20241 | 112.263542 | 16326 | 124.3662302 | 12.10268816 |
| Canovanas | 38051 | 128.8513358 | 9614 | 144.7167748 | 15.86543895 |
| Carolina | 167101 | 105.8878918 | 16318 | 129.9982306 | 24.1103388 |
| Catano | 34726 | 92.72322481 | 366 | 22.28014536 | -70.44307945 |
| Cayey | 33372 | 131.5935674 | 10297 | 141.5451043 | 9.951536893 |
| Ceiba | 8664 | 125.8521574 | 2921 | 144.4873475 | 18.6351901 |
| Ciales | 6506 | 128.1909789 | 9201 | 132.064018 | 3.873039066 |
| Cidra | 35788 | 139.0953767 | 9103 | 144.6419186 | 5.54654186 |
| Coamo | 19555 | 123.491492 | 21305 | 130.1681207 | 6.676628686 |
| Comerio | 10789 | 147.7477107 | 9569 | 147.7308512 | -0.016859503 |
| Corozal | 19214 | 138.0035264 | 16921 | 146.8158799 | 8.812353498 |
| Dorado | 31411 | 119.3032943 | 9593 | 118.2573664 | -1.045927903 |
| Fajardo | 29141 | 129.0982873 | 6103 | 125.6911724 | -3.407114891 |
| Florida | 7397 | 130.7236689 | 4984 | 138.9337151 | 8.210046231 |
| Guanica | 7399 | 122.7786932 | 9106 | 112.0606411 | -10.71805212 |
| Guayama | 26297 | 113.600362 | 17569 | 129.4858553 | 15.88549335 |
| Guayanilla | 7889 | 116.6925782 | 12069 | 120.0988391 | 3.406260844 |
| Guaynabo | 86777 | 93.99245942 | 2966 | 145.3600223 | 51.36756289 |
| Gurabo | 48005 | 125.4824605 | 6773 | 142.3920225 | 16.90956204 |
| Hatillo | 29900 | 110.8136065 | 10645 | 125.5957904 | 14.78218391 |
| Hormigueros | 11880 | 103.1943697 | 3418 | 101.0647723 | -2.129597467 |
| Humacao | 40433 | 147.0883015 | 18263 | 149.4441409 | 2.35583939 |
| Isabela | 35681 | 112.8622022 | 10570 | 109.9023268 | -2.959875434 |
| Jayuya | 6692 | 137.5424224 | 8212 | 144.2809508 | 6.73852837 |
| Juana Diaz | 36662 | 119.1877231 | 17706 | 125.1251987 | 5.937475602 |
| Juncos | 33990 | 126.2863376 | 9051 | 137.6024617 | 11.31612411 |
| Lajas | 10431 | 105.4835687 | 12088 | 111.4173258 | 5.933757114 |
| Lares | 10399 | 115.6534615 | 16180 | 129.6857753 | 14.03231385 |
| Las Piedras | 29814 | 139.0308024 | 9557 | 122.2673613 | -16.76344111 |
| Loiza | 25888 | 121.3718004 | 10458 | 149.0298823 | 27.65808192 |
| Luquillo | 15369 | 122.7930278 | 2439 | 118.448435 | -4.344592821 |
| Manati | 33053 | 123.3134543 | 4382 | 136.3571328 | 13.04367849 |
| Maunabo | 5330 | 159.9292153 | 11283 | 130.4950861 | -29.43412927 |
| Mayagüez | 65738 | 93.75765302 | 4523 | 124.7118345 | 30.95418152 |
| Moca | 27753 | 111.432037 | 5882 | 158.0847407 | 46.65270375 |
| Morovis | 20574 | 150.2495865 | 18525 | 124.0083323 | -26.24125416 |
| Naguabo | 14095 | 140.9158419 | 12662 | 117.9869679 | -22.92887397 |
| Naranjito | 21521 | 142.0828764 | 12581 | 148.5617194 | 6.478843039 |
| Orocovis | 5008 | 147.7942415 | 13806 | 149.7071323 | 1.912890817 |
| Patillas | 9943 | 143.4638872 | 7385 | 143.7484575 | 0.284570244 |
| Penuelas | 16237 | 126.1705301 | 16478 | 144.8707514 | 18.7002213 |
| Ponce | 125586 | 95.81531153 | 7017 | 148.2847711 | 52.46945957 |
| Quebradillas | 17655 | 118.5006902 | 6440 | 127.8921488 | 9.39145856 |
| Rincon | 7007 | 125.0705336 | 25440 | 115.0984009 | -9.97213275 |
| Rio Grande | 39790 | 129.0716944 | 5645 | 112.9568819 | -16.11481254 |
| Sabana Grande | 13204 | 107.0110601 | 5222 | 116.1643198 | 9.153259759 |
| Salinas | 16663 | 128.464211 | 13803 | 140.0236158 | 11.55940475 |
| San German | 16706 | 107.0439748 | 12731 | 112.3670992 | 5.323124411 |
| San Juan | 361270 | 95.4386751 | 14140 | 134.1178636 | 38.6791885 |
| San Lorenzo | 24612 | 132.6912846 | 20055 | 115.3004773 | -17.39080723 |
| San Sebastian | 18989 | 110.1419897 | 2190 | 116.7552635 | 6.613273856 |
| Santa Isabel | 16552 | 124.9232071 | 13804 | 154.635638 | 29.71243089 |
| Toa Alta | 65305 | 122.5974659 | 22518 | 117.8079334 | -4.78953252 |
| Toa Baja | 79324 | 115.4737736 | 6450 | 132.810766 | 17.33699245 |
| Trujillo Alto | 66902 | 117.602951 | 6310 | 141.5851829 | 23.98223193 |
| Utuado | 9213 | 124.882041 | 6476 | 122.2496927 | -2.63234835 |
| Vega Alta | 28022 | 132.8565161 | 4152 | 129.6671893 | -3.18932684 |
| Vega Baja | 45759 | 125.2537037 | 22767 | 133.0629797 | 7.80927592 |
| Villalba | 16426 | 135.4018488 | 14352 | 133.0666466 | -2.335202215 |
| Yabucoa | 15868 | 159.3540528 | 10568 | 136.9246289 | -22.42942398 |
| Yauco | 29484 | 111.2906535 | 8672 | 138.9104155 | 27.61976205 |
