# Supplementary figures and images for: Satellite-based assessment of electricity restoration efforts in Puerto Rico after Hurricane Maria
Source: PLoS One. 2019 Jun 28;14(6):e0218883. doi: 10.1371/journal.pone.0218883 (PMC6599127; doi:10.1371/journal.pone.0218883)

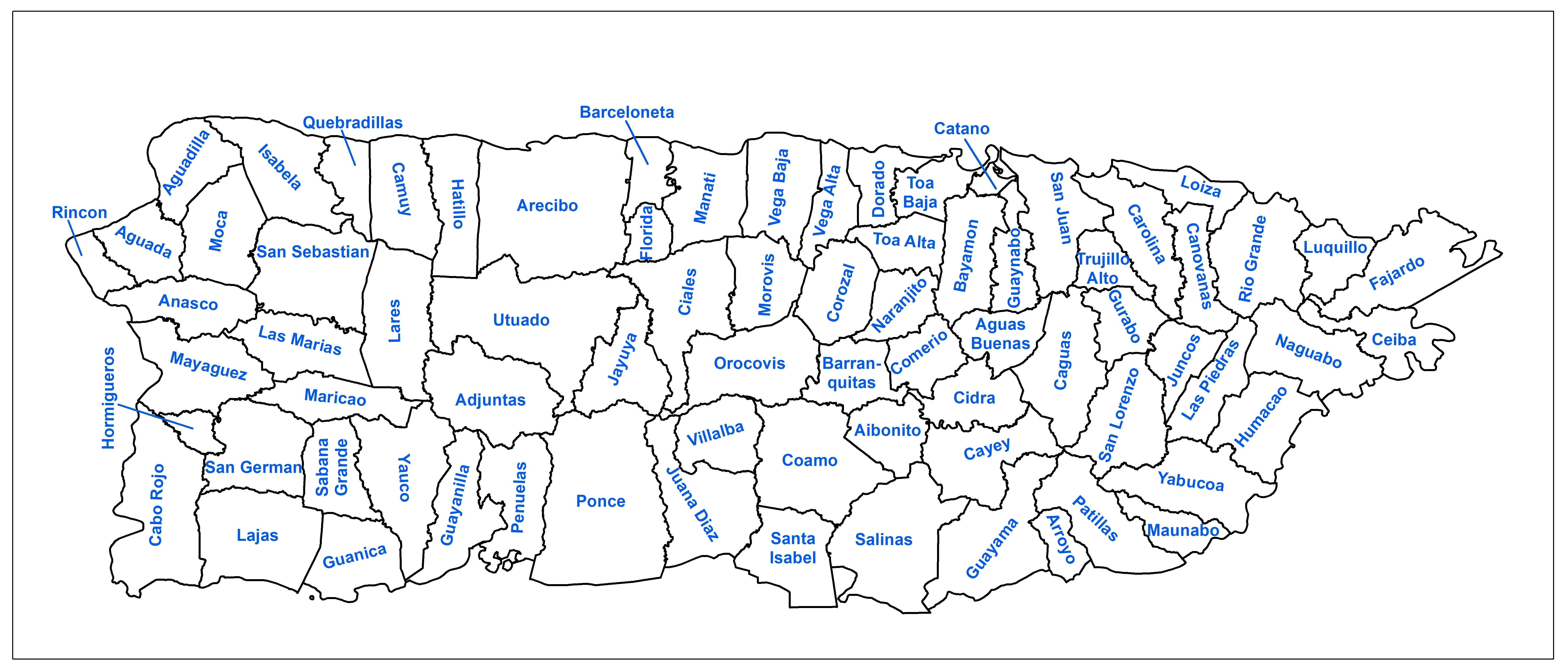

Supplement: S1 Fig — While Hurricane Maria weakened slightly before making landfall on September 20 in southeastern Puerto Rico, the storm’s track (shown in S2 Fig) was a near worst-case scenario. It ripped directly across the island with sustained winds of 155 miles per hour (250 kilometers per hour). All of the municipalities in Puerto Rico saw outages after the storm. (TIFF) [file pone.0218883.s002.tiff]

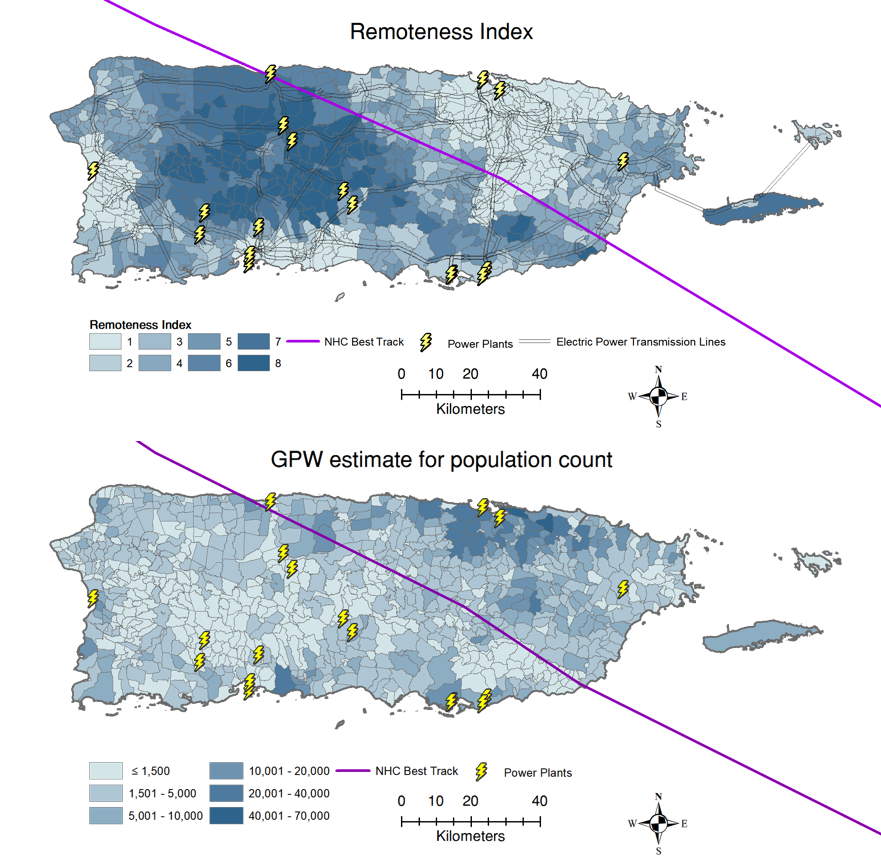

Supplement: S2 Fig — (A) Population count estimates derived from Gridded Population of the World (GPW) collection 4 products. The National Hurricane Center (NHC) track of Hurricane Maria is shown as a purple line. (B) Heatmap of average travel time to population centers of at least 50,000 individuals using local road networks across Puerto Rico–adapted from Kishore et al. (2018) (4). (TIFF) [file pone.0218883.s003.tiff]

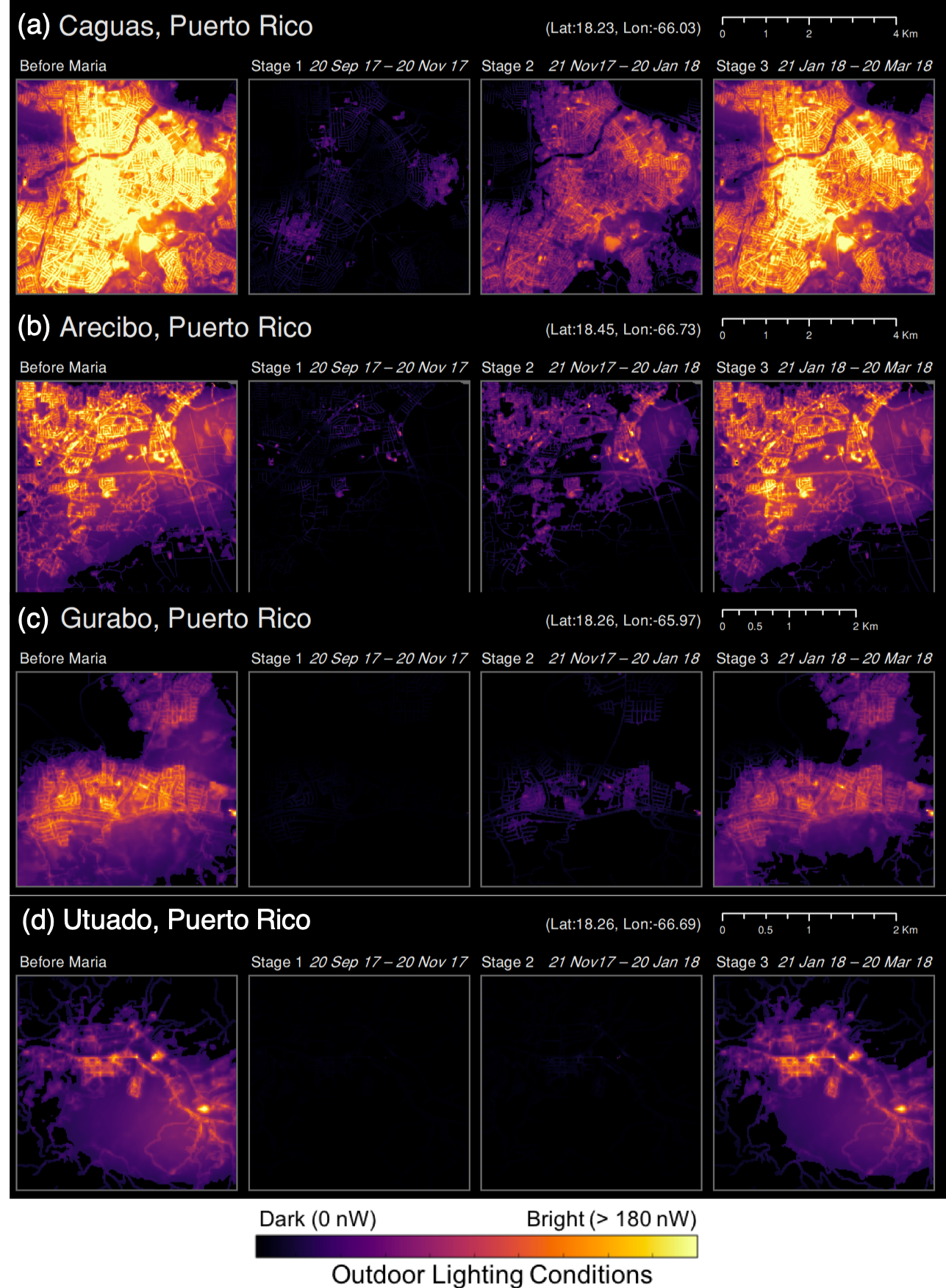

Supplement: S3 Fig — NASA Black Marble high definition nighttime lights subsets show recovery of electricity before Maria and at each state of recovery in, e.g. (A) Caguas (Population = 134,481) and (B) Arecibo (Population = 94,658), and adjacent rural towns e.g., (C) Gurabo (Population = 46,406) and (D) Utuado (Population = 32,776). (TIFF) [file pone.0218883.s004.tiff]

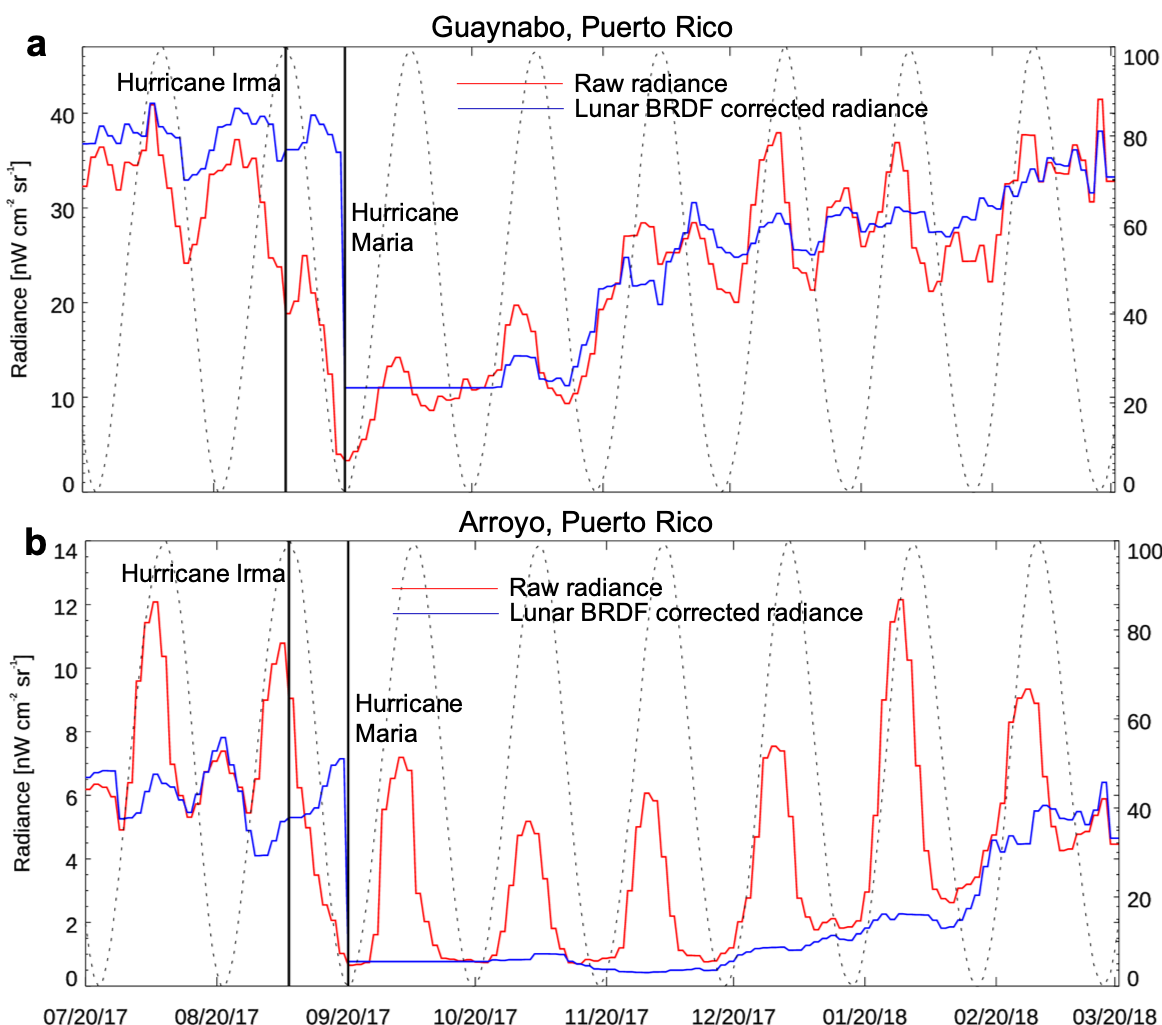

Supplement: S4 Fig — The standard 500-meter Black Marble product (VNP46) is shown to reduce noise, particularly by removing artifacts stemming for lunar reflectance anisotropy effects. The original at-sensor cloud-corrected VIIRS Day/Night Band radiances are shown as red lines (units of nW·cm-2·sr-1). Those resulting from NASA’s Black Marble nighttime lights product are shown as blue lines. The vertical lines correspond to the passing of Hurricane Irma and Maria, respectively. The trailing dotted black line tracks the lunar phase. (TIFF) [file pone.0218883.s005.tiff]
